# Lymphedema management in patients with head and neck cancer: a systematic review of randomized controlled trials on physical therapy interventions

**DOI:** 10.1007/s00520-025-09438-1

**Published:** 2025-04-26

**Authors:** Laura de-la-Cruz-Fernández, Noelia Galiano-Castillo, Pilar Galván-Banqueri, Eduardo Castro-Martín, Mario Lozano-Lozano, Paula Postigo-Martin, Maria Lopez-Garzon

**Affiliations:** 1 Huétor Salud Fisioterapia, Granada, Andalucía, Spain; 2 Biomedical Group (BIO277), Department of Physical Therapy, Health Sciences Faculty, University of Granada, Granada, Spain; 3 Instituto de Investigación Biosanitaria Ibs. GRANADA, Granada, Spain; 4 Sport and Health Research Center (IMUDs), Parque Tecnológico de La Salud, Granada, Spain; 5 Department of Radiation Oncology, Hospital Universitario Virgen de Las Nieves, Granada, Spain

**Keywords:** Head and neck neoplasms, Intermittent pneumatic compression devices, Lymphedema, Physical therapy modalities, Systematic review

## Abstract

**Purpose:**

Lymphedema is one of the most common side effects following oncological treatment. This systematic review analyzed the latest literature concerning the efficacy of physical therapy interventions in treating secondary lymphedema in patients with head and neck cancer.

**Methods:**

Medline, Web of Science, Scopus, and Cochrane Library were searched for studies published before August 2023. Randomized controlled trials in which physical therapy was applied to treat lymphedema in head and neck cancer were included. Reviewers blinded screened the articles retrieved, scored methodological quality, and extracted data. The review was conducted according to the PRISMA statement and registered in PROSPERO (CRD42023439643). Risk of bias assessment was performed using the Cochrane tools.

**Results:**

A total of four randomized controlled trials were included. They comprise 167 patients, and only one of the studies achieved a low risk of bias. Interventions were kinesio taping, compression therapy, manual lymphatic drainage and/or exercise applied in combination with skin care and self-management. Some adverse effects related to intervention were mild and transitory.

**Conclusion:**

The findings shown by this review were that an exercise program plus manual lymphatic drainage supplemented with kinesio taping or compression therapy could be beneficial for external lymphedema. Neither therapy achieved an improvement in internal lymphedema.

**Supplementary Information:**

The online version contains supplementary material available at 10.1007/s00520-025-09438-1.

## Introduction

Lymphedema is one of the most common side effects following oncological treatment for head and neck cancer (HNC) and is the most frequently underdiagnosed condition [1]. The incidence of lymphedema secondary to HNC is highly variable in the scientific literature, with reported values ranging from 10 to 98 [2–4]. This condition could be secondary to obstruction (tumor), resection (surgery), or deterioration (radiotherapy) of the lymphatic drainage channels, which can affect external structures (soft tissues of the face and neck) or internal structures (oral cavity, pharynx, and larynx) [5]. Thus, as side effects related to both cancer and treatment, lymphedema can cause adverse physical outcomes in this oncology population such as airway compromise, difficulty vocalizing, decreased cervical range of motion, abnormal head posture, musculoskeletal discomfort, and swallowing problems [6–9]. However, head and neck lymphedema (HNL) is not only associated with physical damage, as it often causes swelling and visible changes in the treated areas which can affect their overall body image and self-esteem [10].

The combination of these emotional and physical effects can significantly affect patients’ quality of life [11], impacting their overall well-being and ability to lead a normal, active life [12].

Early detection and effective management strategies are essential in addressing this limiting condition [13], and its consequences on patients with HNC [14], which could reduce the health care costs associated with such sequelae [10, 15, 16]. In 2023, Mullan et al. [17] conducted a systematic review on the management of chronic HNL, determining that effective interventions are in their early stages, highlighting the need for research on them.

Rehabilitation strategies, in particular physical therapy, can revolve around complete decongestive therapy (CDT) as the current standard of care. CDT includes manual lymphatic drainage (MLD), short stretch compression bandaging, compression garments, exercises targeting the face, neck, and oral cavity, and proper skincare [18]. There is speculation that CDT may improve HNL-related dysphagia [19] and despite limited data, research suggests that patients with HNC respond positively to CDT [20], and a recent randomized controlled trial (RCT) has shown promising results with CDT and home-based programs, with CDT being more effective [21]. Advanced pneumatic compression devices (APCDs) have also demonstrated preliminary efficacy and safety in HNL [22]. Other techniques, such as the application of kinesio taping with lymphatic drainage parameters, shockwave therapy, photobiomodulation therapy, low-frequency electrotherapy, or the use of deep oscillation therapy complementary to MLD, are also beginning to be used successfully to treat HNL [23–25]. However, unfortunately, Tyker et al. 2019 [20] established that there is no consensus on the most effective type, duration, and parameters of physical therapy to address this issue.

To the best of our knowledge, a previous review proposed by Tyker [20] includes different modalities of physical therapy for managing HNL, although the results found are limited to CDT alone, excluding all the other therapies mentioned above. In addition, this review [20] includes not only RCTs but also single-arm, single-case, observational studies, whose methodological quality was not assessed in any case. Another limitation of this review is the lack of homogeneity in terms of the main outcome studied, which in some cases was pain and in others was lymphedema or functionality. Finally, the synthesis of Tyker et al. [20] covers studies published between 1965 and 2018, thus leaving the information outdated due to the recent publication of new studies. More recently, Cheng and colleagues [26] have appraised comprehensive English-based evidence for rehabilitation interventions in this population, including different kinds of designs, assuming that authors have based their findings mainly on nonrandomized controlled trials (74%); although the conclusions are valuable and pertinent, the results could be considered a mixture of data that may make readers draw unclear conclusions without scientific rigor. Finally, in comparison to the recent systematic review by Mullan et al. [17], there are several limitations that need to be addressed. Mullan et al.’s review [17], while comprehensive included a heterogeneous mix of study designs, as did the study of Chen and colleagues [26]. Furthermore, their review highlighted a critical issue of poor patient adherence to interventions, undermining the real-world applicability of their results. Additionally, Mullan et al. [17] did not provide an in-depth analysis of specific physical therapy techniques like CDT, kinesio taping, and APCDs, which are crucial in the current therapeutic landscape. These limitations underscore the need for a more focused and methodologically rigorous review to provide clearer and more actionable insights into the management of secondary lymphedema in patients with HNC. As a result, this study aimed to systematically analyze the latest literature concerning the efficacy of physical therapy interventions in treating secondary HNL.

## Material and methods

The methodology and data presentation of this systematic review were carried out following the guidelines established by the PRISMA (Preferred Reporting Items for Systematic Reviews and Meta-Analyses) statement [27]. Based on the PICOS strategy (population, intervention, comparison, outcomes, and design of studies) [28], the following research question was formulated: Are the different physical therapy interventions used for the management of secondary HNL effective?

This study was submitted and accepted in the International Prospective Register of Ongoing Systematic Reviews (PROSPERO) with the following registration code: CRD42023439643. Protocol was not prepared.

### Search strategies

The research question described was used in a structured search carried out in the following databases: Medline (PubMed), Web of Science, Scopus, and Cochrane Library. This search, whose language was Spanish or English, was carried out during the period from June to August 2023.

The MeSH terms used in the search formulas were the following and always in accordance with the PICOS strategy (Table 1): “Head and Neck Neoplasms” for the target population (*P*), “Physical Therapy Modalities” for the intervention (I), “Lymphedema,” as the main outcome evaluated by circometry, endoscopy, ultrasound, and scales (O) and “Randomized Controlled Trials,” which delimits the design (S), together with their corresponding Entry Terms and using the Boolean operators AND/OR. Online Resource 1 presents the search formula used in Medline.

**Table 1:** PICOS (search criteria)

	Inclusion criteria	Exclusion criteria
P	Patients with head and neck cancer	Patients with lymphedema caused by a different type of cancer
I	Physical therapy modalities	NA
C	Indifferent	NA
O	Lymphedema	NA
S	RCT	NA

*C*, comparison; *I*, intervention; *NA*, not applicable; *O*, outcomes; *P*, population; *RCT*, randomized controlled trial; *S*, study design

### Study selection

The systematic selection of studies was carried out in three phases. In the first phase, studies that were duplicated in the different databases were manually eliminated. In the second phase, the remaining studies were analyzed and selected by title and abstract, excluding those that did not meet the eligibility criteria. Finally, in the last phase, the selected studies were read and analyzed in full text. During the screening of studies by title and abstract, a peer review was performed involving two reviewers. The quantification of the agreement between reviewers was performed by calculating the Kappa Index [29].

### Risk of bias assessment

Because one of the inclusion criteria was RCT design, the risk of bias (RoB) of each article was independently and critically appraised using the Cochrane Risk of Bias tool RoB 2 by two different blinded reviewers [30]. Reviewers decided that assessments for one of the RoB domains, “Bias due to deviations from intended interventions,” were aimed to quantify the effect of adhering to the interventions as specified in the trial protocol (the “per-protocol effect”).

Specifically, we assessed trial participants’ nonadherence to their assigned intervention. Disagreements between reviewers were resolved by a third external reviewer.

### Data extraction

Once the studies were selected, the most relevant data were extracted from each of them and were included in a summary table (Excel spreadsheet). The information collected from the different studies included the name of the first author, year of publication, study design, sample size, and population, type of intervention, outcome, results of the intervention in each of the groups, and possible adverse effects (AEs) if indicated. Finally, the quality of the chosen databases was determined by sensitivity/precision analysis (Online Resource 2).

## Results

### Selection of studies

**Fig. 1:**
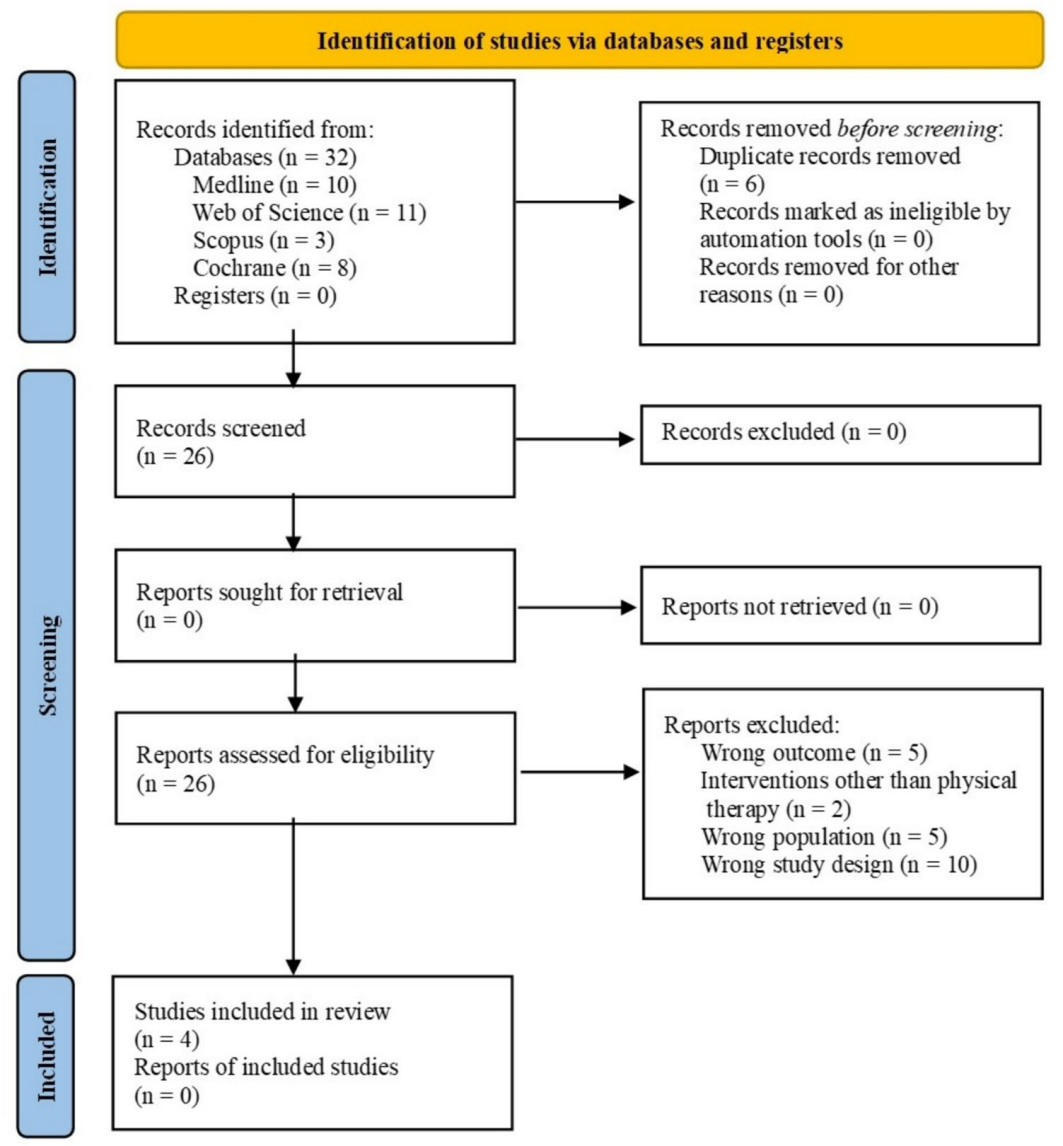
Flow chart of the study selection procedure

A total of 32 studies were identified, of which 10 belonged to Medline, 11 to Web of Science, three to Scopus, and eight to Cochrane Library. Of these, six were excluded because they were duplicates, and 26 studies were screened by title and abstract. Finally, four studies were selected for final full-text review. The process of screening and selection of the studies is shown in Fig. 1.

During the screening of the studies by title and abstract, a peer review was performed. Initially, the percentage of agreement between the two reviewers, based on the Kappa index, was 96.15%, and thus, the concordance was almost perfect [29]. After discussion between the two reviewers, 100% agreement was reached.

### Descriptive analysis

The main characteristics and results of each of the 4 RCTs selected for this systematic review are summarized in Table 2.

**Table 2:** Main characteristics and results of the studies analyzed

Author year	Study design	Sample size and population	Intervention CG	Intervention EG	Outcome (lymphedema)	Results	Adverse effects
Atar et al. 2022	RCT	*N* = 58 Patients diagnosed with secondary HNL longer than 3 months who had undergone surgery with unilateral/bilateral neck dissection and radiotherapy (with or without chemotherapy) • CG = 28 • EG = 30	4 weeks→ Home exercise program (15 repetitions of each exercise 1/day) + MLD (45 min 5 days/week the first week and 2 days/week the remaining 3 weeks) + non-therapeutic kinesio taping (does not follow placement parameters) 4 weeks follow-up→ continue with the home exercise program	4 weeks→ Home exercise program (15 repetitions of each exercise 1/day) + MLD (45 min, 5 days/week the first week and 2 days/week the remaining 3 weeks) + therapeutic kinesio taping (placement using the lymphatic technique) 4 weeks follow-up→ continue with the home exercise program	External lymphedema (MDACC-HNL → tape measurements of facial circumference, facial point to point, neck circumference and total circumference landmarks) Stage of lymphedema (MDACC-HNL Scale) Internal lymphedema (Endoscopy + Patterson Edema scale)	Baseline vs end of intervention: External lymphedema *Between-group effects:* • Facial circumference: EG > CG (*P* = 0.032; ES = 0.39) • Facial point to point: EG = GC (*P* = 0.237; ES = 0.124) • Neck circumference: EG > CG (*P* = 0.001; ES = 0.688) • Total circumference: EG > CG (*P* = 0.002; ES = 0.513) *Within-group effects:* NR Stage of lymphedema *Between-group effects:* NR *Within-group effects:* • EG ↑ (*P* = 0.001) ○ Stage 2 (13% → 0%) ○ Stage 1b (70% → 17%) ○ Stage 1a (17% → 83%) • CG ↑ (*P* = 0.001) ○ Stage 2 (12% → 4%) ○ Stage 1b (76% → 32%) ○ Stage 1a (12% → 64%) Internal lymphedema *Between-group effects:* • EG = CG (*P* = 0.890) *Within-group effects:* NR End of intervention vs follow-up: External lymphedema *Between-group effects:* NR *Within-group effects:* NR Stage of lymphedema *Between-group effects:* NR *Within-group effects:* • EG ↑ (*P* = 0.001) ○ Stage 1b (17% → 7%) ○ Stage 1a (83% → 93%) • CG ↑ (*P* = 0.001) ○ Stage 2 (4% → 0%) ○ Stage 1b (32% → 32%) ○ Stage 1a (64% → 68%) Internal lymphedema *Between-group effects:* • EG = CG (*P* = 0.860) *Within-group effects:* NR	Mild (grades 1–2): skin irritation, slight itching and rashes (related to the application of kinesio taping)
Ozdemir et al. 2021	RCT	*N* = 21 patients diagnosed with lymphedema secondary to HNC currently in remission (for at least 3 months) and that had undergone surgery with unilateral/bilateral neck dissection and received chemo and/or radiotherapy • CG = 7 • EG_1_ = 7 (CDT) • EG_2_ = 7 (home program)	4 weeks→ No intervention. Medical check-ups without physiotherapeutic intervention or counseling	EG_1_ → 4 weeks: 30 min of MLD (1/day and 5 days/week) + compression mask at least 4–6 h/day + skin care + neck, face, tongue, and postural exercises while wearing the compression mask (10 repetitions of each exercise 1/day and 5 days/week) EG_2_ → 4 weeks: self-administered lymphatic drainage on several face and neck areas (1/day) + neck, face, tongue and postural exercises (10 repetitions of each exercise 1/day)	External lymphedema (MDACC-HNL → tape measurements of facial circumference, facial point to point, neck circumference, and total circumference landmarks) External lymphedema volume (3D scanning of face and neck surface with Artec Eva 3D scanner) Stage of lymphedema (MDACC-HNL Scale)	At the end of intervention: External lymphedema *Between-group effects:* • Facial circumference: EG _1–2_ = CG (*P* = 0.701) • Facial point to point: EG _1–2_ = CG (*P* = 0.228) • Neck circumference: EG _1–2_ = CG (*P* = 0.935) • Total circumference: EG _1–2_ = CG (*P* = 0.625) *Within-group effects:* • Facial circumference: ○ EG_1_ ↔ (*P* > 0.05) ○ EG_2_ ↔ (*P* > 0.05) ○ CG ↓ (*P* < 0.05) • Facial point to point: ○ EG_1_ ↑ (*P* < 0.05) ○ EG_2_ ↑ (*P* < 0.05) ○ CG ↔ (*P* > 0.05) • Neck circumference: ○ EG_1_ ↔ (*P* > 0.05) ○ EG_2_ ↔ (*P* > 0.05) ○ CG ↔ (*P* > 0.05) • Total circumference: ○ EG_1_ ↑ (*P* < 0.05) ○ EG_2_ ↔ (*P* > 0.05) ○ CG ↔ (*P* > 0.05) External lymphedema volume (3D scan) *Between-group effects:* • EG _1–2_ = CG (*P* = 0.723) *Within-group effects:* NR Stage of lymphedema *Between-group effects:* NR *Within-group effects:* • EG_1_ ↑ (*P* = 0.014) ○ Stage 2 (14.3% → 0%) ○ Stage 1b (71.4% → 14.3%) ○ Stage 1a (14.3%→ 85.7%) • EG_2_ ↑ (*P* = 0.830) ○ Stage 2 (14.3% → 0%) ○ Stage 1b (28.6% → 14.3%) ○ Stage 1a (57.1% 85.7%) • CG ↔ (*P* = 0.998) ○ Stage 2 (28.6% → 28.6%) ○ Stage 1b (28.6% → 28.6%) ○ Stage 1a (42.9% → 42.9%)	None
Ridner et al. [22]	RCT	*N* = 43 patients with a clinical diagnosis of lymphedema secondary to HNC currently in remission • CG = 24 • EG = 19	8 weeks→ usual care: lymphedema self-care learning program (skin and postural care, gentle exercises, and compression) + self-care tools (diary, self-care checklists and a record of upcoming medical appointments)	8 weeks→ usual care + APCD (pressotherapy) 2 times a day (23–45 min per application)	Subjective perception of lymphedema symptoms (LSIDS-HN and VHNSS plus GSS v2.0 Questionnaires) External lymphedema (visual inspection -photographs of head and neck- and palpation) Internal lymphedema (Endoscopy + Modified Patterson Scale: a grade of normal, mild, moderate, or severe was documented for each site or space)	At the end of intervention: LSIDS-HN *Between-group effects:* • Soft tissue EG > CG (*P* = 0.004; ES: 0.86) • Neurological symptom EG > CG (*P* = 0.047; ES: 0.60) • Other clusters EG = CG (*P* > 0.05) *Within-group effects:* NR VHNSS-GSS *Between-group effects:* • Swallowing solids EG > CG (*P* = 0.016; ES = 0.8) • Mucous related symptoms EG > GC (*P* = 0.05; ES = 0.57) • ↓Pain GE < CG (*P* = 0.008; ES = 0.89) • Other clusters EG = CG (*P* > 0.05) *Within-group effects:* NR External lymphedema *Between-group effects:* • Front view EG > CG (*P* < 0.001; ES = 1.26) • Right view EG > CG (*P* = 0.004; ES = 0.96) • Left view EG > CG (*P* = 0.005; ES = 0.84) *Within-group effects:* • Front view: ↑24% EG vs ↓5% CG • Right view: ↑22% EG vs ↑7% CG • Left view: ↑17% EG vs ↑4% CG Internal lymphedema *Between-group effects:* • % of visible sites with swelling EG = CG (*P* = 0.961; ES = 0.01) • Severity of the swelling EG = CG (*P* = 0.948; ES = 0.02) *Within-group effects:* NR	Severe (grade 3–5): cellulitis, stroke, hyponatremia and death (not related to the intervention) Mild (grade 1–2): erythema, oedema, ecchymosis, tenderness, numbness and lumps (these AEs were common)
Tsai et al. 2022	RCT	*N* = 39 patients with oral cavity cancer • CG = 20 • EG = 19	Until discharge → 30-min exercise sessions (breathing exercises, neck and shoulder mobilizations, and coughing)	Until discharge→ 30-min exercise sessions (breathing exercises, neck and shoulder mobilizations, and coughing) + 30 min MLD	External lymphedema. skin-to-bone distance (US at 6 sites on face and neck) Stage of lymphedema (Földi and Miller scales) Facial distances (tape measurements)	At the end of intervention: External lymphedema. Skin-to-bone distance (ultrasound) *Between-group effects:* • Ascending mandibular ramus right side EG > CG (*P* < 0.0001) • Horizontal mandible right side EG > CG (P < 0.0001) *Within-group effects:* • Ascending mandibular ramus right side: EG ↑ (*P* < 0.05) • Horizontal mandible right: EG ↑ and CG ↑ (*P* < 0.05) • Ascending mandibular ramus left side: EG ↑ and CG ↑ (*P* < 0.05) • Horizontal mandible left side: EG ↑ and CG ↑ (*P* < 0.05) Stage of lymphedema *Between-group effects:* • Miller: EG = CG (*P* = 0.987) • Földi: EG = CG (*P* = 0.172) *Within-group effects:* • Miller: ○ EG ↔ (P > 0.05) ○ CG ↔ (P > 0.05) • Földi: ○ EG ↔ (P > 0.05) ○ CG ↔ (P > 0.05) Facial distances *Between-group effects:* • For both sides EG = GC (P = 0.224) *Within-group effects:* • For right side EG ↑ and CG ↑ (P < 0.001)	None

*AE*, adverse effects; *APCD*, advanced pneumatic compression devices; *CDT*, complex decongestive therapy; *CG*, control group; *EG*, experimental group; *ES*, effect size; *HNL*, head and neck lymphedema; *LSIDS-HN*, lymphedema symptom intensity and distress survey-head and neck; *MDACC-HNL*, MD Anderson Cancer Center-Head and Neck Lymphedema*; MLD*, manual lymphatic drainage; *NR*, not reported; *RCT*, randomized controlled clinical trial; *US*, ultrasound; *VHNSS-GSS*, Vanderbilt Head and Neck Symptom Survey plus General Symptom Survey; ↑, improvements in the outcome; ↓, worsening in the outcome; ↔, maintenance of the outcome.

The total number of participants was 161, of whom 79 were in the control group (CG) and 82 were in the experimental group (EG). Of these, 87 were male (53.89%), 35 were female (22.75%), and 39 were unspecified (23.35%), as Tsai et al. 2022 [31] did not include data about the sex of the patients. The sample size ranged from 21 to 58 participants, and the age range ranged from 36.2 to 70.5 years.

The main regions of HNC were the oral cavity, larynx, oropharynx, and salivary and endocrine glands. All patients underwent some type of surgery as the main oncological treatment, receiving additional radiotherapy in 43.1% of the cases and chemotherapy in 10.1%. On examining the data referring to the stage of lymphedema in which the patients were at the beginning of the studies, we found 22% in stage Ia, 56.7% in stage Ib, 13.2% in stage II, and 8% in stage III; excluding the data of the participants in the study by Ridner et al. [22] as this information was not collected. All of the RCTs included had one CG and one EG, except for one, which had two EGs [21].

Regarding the type of intervention, one of them was based on the use of an APCD (pressotherapy) complemented with the usual care [22]; the other three interventions used the combination of an exercise program (all similar to each other) together with MLD [21, 31, 32]. Additionally, one of the interventions also included the application of compressive therapy [21], while another included the application of therapeutic kinesio taping [32].

For the CGs, the treatment was heterogeneous, one consisting of the usual self-care learning treatment for lymphedema [22], another comprised medical check-ups without physical therapy intervention or counseling [21] and the other two CGs participated in an exercise program [31, 32], one of them involving MLD and the application of kinesio taping with nontherapeutic parameters [32].

The duration of the sessions, as well as their frequency, varied greatly among the different studies. The period during which the intervention was prolonged ranged from 4 [21, 32] to 8 weeks [22]. In the study of Tsai et al. 2022 [31], the intervention was performed until the patients were discharged, with an average of eight sessions in the CG and ten in the EG. Moreover, only the study by Atar et al. in 2022 [32] included an additional 4-week follow-up period after the end of the intervention.

### Qualitative analysis

Lymphedema was the primary outcome in this systematic review and was assessed through different tools in the included RCTs. All studies assessed external lymphedema using the MDACC-HNL scale [21, 32], visual inspection (photographs) and palpation [22], ultrasound [31], and 3D scanning of the face and neck surface using an Artec Eva 3D scanner [21]. On the other hand, two of the studies assessed internal lymphedema using endoscopy and the Patterson Edema scale [22, 32].

In addition, three of the studies assessed the stage of lymphedema via the MDACC-HNL scale [21, 32] and the Földi and Miller scales [31]. Finally, only one of the studies evaluated the subjective perception of lymphedema symptoms using the Lymphedema Symptom Intensity and Distress Survey-Head and Neck (LSIDS-HN) and the Vanderbilt Head and Neck Symptom Survey plus General Symptom Survey (VHNSS-GSS) [22].

Regarding the results between groups, in the study by Atar et al. [32], statistically significant differences were found in favor of the EG for the external lymphedema variable, specifically regarding facial, neck, and total circumferences (*p* = 0.032, *p* = 0.001, *p* = 0.002, respectively). However, no statistically significant differences were found for the rest of the variables neither after the intervention nor for any of the variables evaluated after the 4-week follow-up. Second, Ozdemir et al. [21] found no statistically significant differences after the intervention for any of the variables. On the other hand, Ridner et al. [22] found significant differences in the EG in the soft tissue and neurological tissue subscales of the LSIDS-HN questionnaire (*p* = 0.004, *p* = 0.047, respectively) and in the swallowing solids, mucus-related symptoms and pain subscales of the VHNSS-GSS questionnaire (*p* = 0.016, *p* = 0.05, *p* = 0.008, respectively). Similarly, statistically significant differences were found in favor of the EG for external lymphedema affecting frontal (*p* < 0.001), right lateral (*p* = 0.004), and left lateral vision (*p* = 0.005). However, for internal lymphedema, no significant differences were found after the intervention. Finally, Tsai et al. [31] described statistically significant differences for the variables skin-to-bone distance in the ascending mandibular ramus right side (*p* < 0.0001) and in the horizontal mandible right side (*p* < 0.0001) but not for the remaining variables measured.

### Risk of bias of included studies

**Fig. 2:**
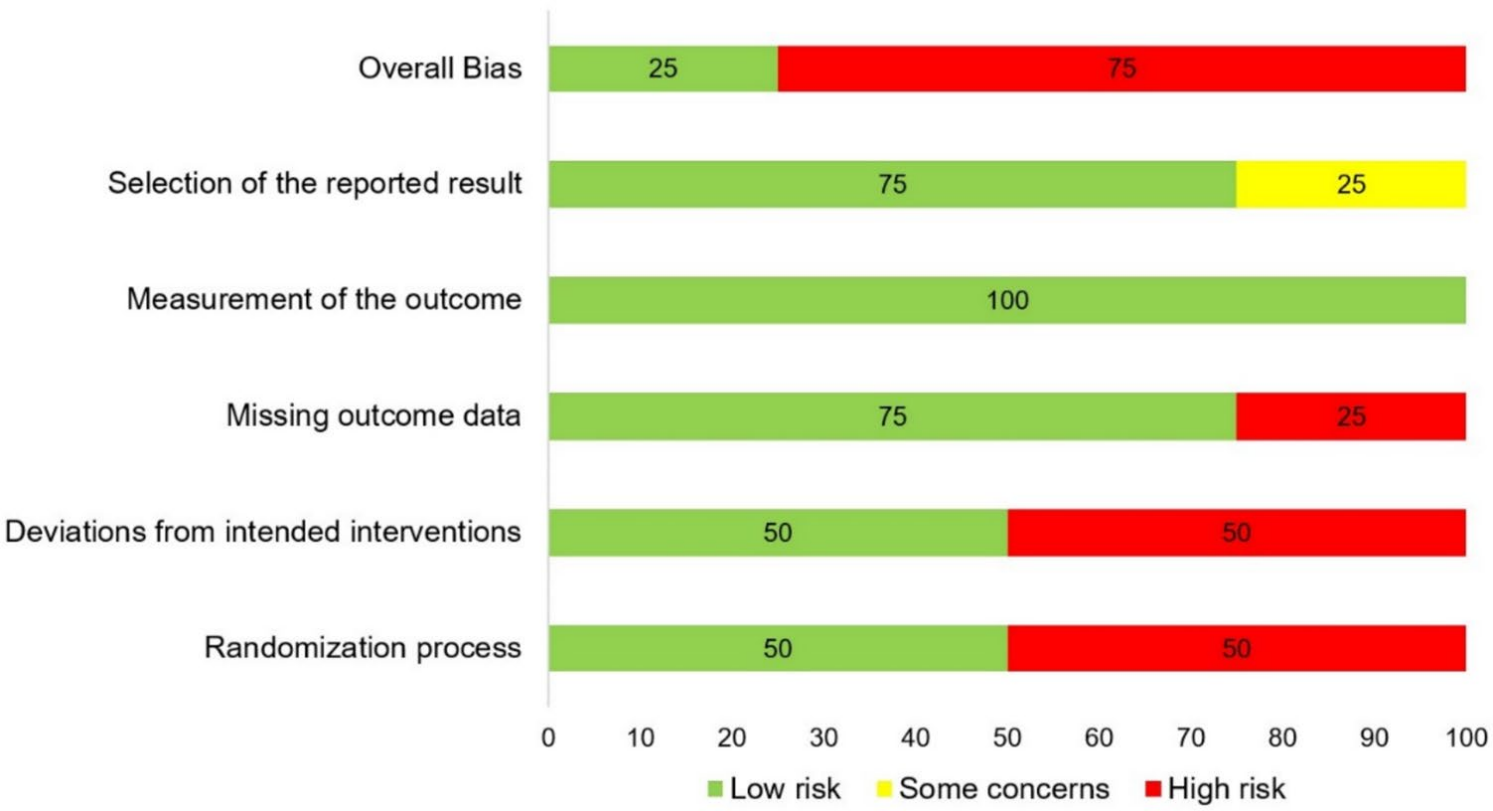
Risk of bias assessment

**Fig. 3:**
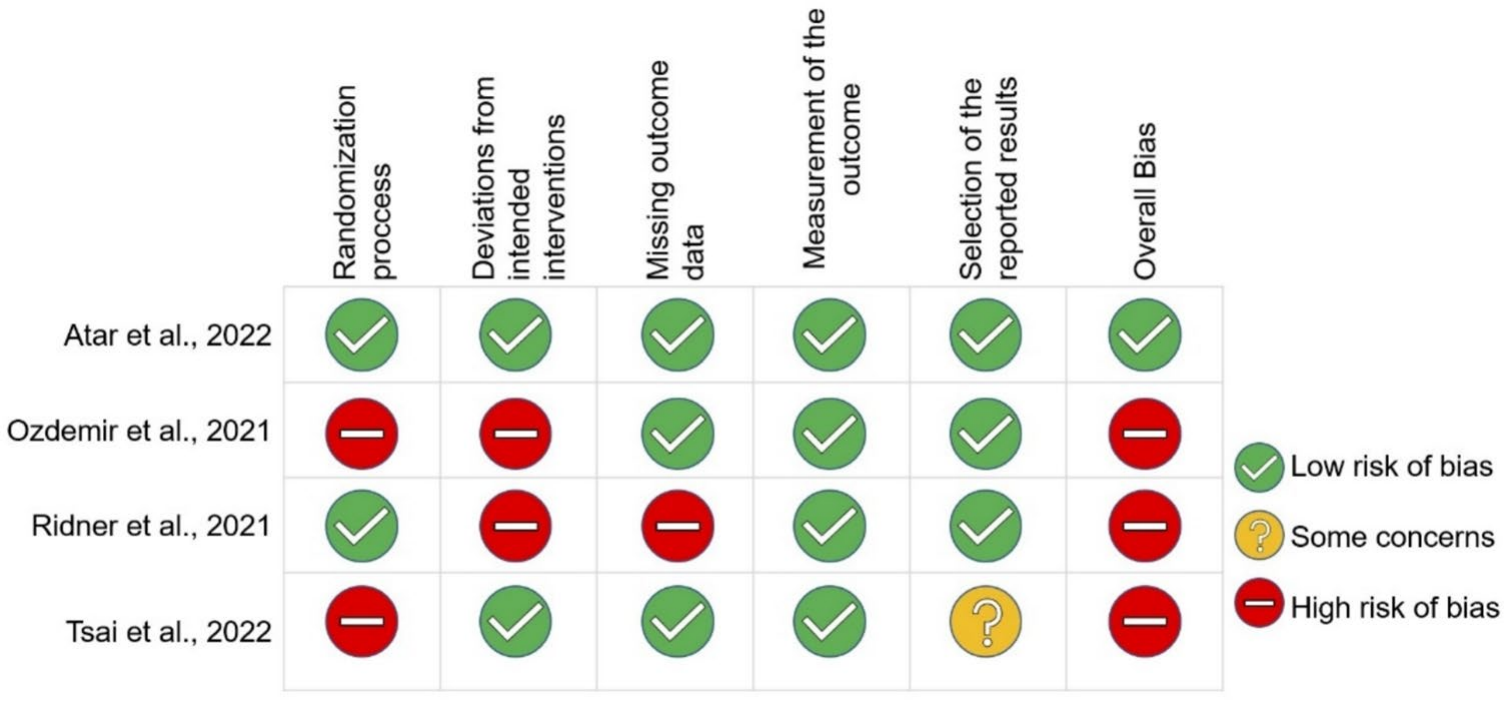
Risk of bias summary of the four included studies

The results of the RoB assessment of the four included RCTs are presented in Fig. 2, and a summary for each study is shown in Fig. 3.

All the studies achieved a low RoB for measurement of the outcome. The main methodological quality issues were the randomization process and deviations from intended interventions, which presented high risk (50%). Similarly, a high risk (25%) was reported for missing outcome. Only one of the studies achieved a low overall RoB [32].

## Discussion

Based on the recognition that early identification of HNL is crucial to prevent disease progression [33], this systematic review explored the current scientific literature with the aim of determining the effectiveness of different physical therapy interventions for the management of secondary HNL. The main findings show that (1) a home exercise program plus MLD and supplemented with kinesio taping [32], (2) and APCD plus usual care [22], and (3) a MLD plus exercise protocol [31] could be beneficial for the treatment of external lymphedema (but not for internal lymphedema). However, self-directed MLD together with unsupervised self-exercises [21] do not seem to show positive results for the treatment of external lymphedema. In comparison to the recent systematic review by Mullan et al. (2023), our study provides a significantly enhanced and updated perspective on the management of secondary HNL through physical therapy interventions. In contrast, our review focuses exclusively on high-quality RCTs, ensuring robust and reliable findings. Our evaluation of AEs related to interventions also offers a comprehensive understanding of the safety profiles of these therapies. Therefore, our review not only addresses the limitations and gaps in Mullan et al.’s work but also contributes substantially to the existing literature with a rigorous and focused approach.

First, in 2022, Atar et al. [32] found a significant reduction in external lymphedema volume by applying kinesio taping with a drainage technique complementary to an exercise program and MLD. In fact, previous studies have already suggested that kinesio taping could be a substitute therapy for the compression normally used (multilayer compression bandage) as part of CDT in cases of low tolerance to these treatments, obtaining similar [34] or even better effects due to its easy application, longer duration, and greater tolerance by patients [35]. Furthermore, Pekyavas et al. [36] found more positive effects when using kinesio taping concomitantly with compression stockings or multilayer bandaging, as the effects of MLD performed as part of CDT were maintained not only during the treatment but also afterward. This may be because the application of kinesio taping with a technique aimed at lymphatic drainage seems to cause a slight elevation of the epidermis, increasing the volume of the dermis containing the lymphatic vessels and thus promoting drainage [34, 36]. Thus, kinesio taping applied with a lymphatic technique in the EG could have allowed us to maintain the effects achieved with the multimodal program of exercises and MLD performed by these patients over a certain period [32]. The kinesio taping application method followed by Atar et al. [32] consists of cutting the kinesio tape in the shape of an octopus, applying without stretching the uncut part, while the strips should be placed by stretching the kinesio tape slightly (5–25%) and orienting it toward the center of the edema [32]. It should be noted that although the patients in this study did not receive treatment immediately after surgery, in the systematic review by Hormann et al. in 2020 [37], kinesio taping had already been shown to be effective for the treatment of edema in patients treated postoperatively.

Second, it is worth highlighting the effectiveness found in the 2021 study conducted by Ridner et al. [22] on the reduction of external lymphedema volume and the improvement of subjective symptoms perceived by the patient through the use of APCD in combination with usual care after only 8 weeks of treatment. In 2014, Zaleska et al. [38] studied the effects of pneumatic compression applied daily for 3 years and observed a reduction in the volume of lymphedema, although it stabilized after the first year of application [38]. In the opinion of these authors, the decrease in volume and pressure of the moving tissue fluid could avoid stress on fibroblasts and keratinocytes, and improve cellular nutrition, avoiding fibrosis. However, there is evidence that lymphatic fluid drainage by pneumatic compression does not correlate with the evacuation of macromolecules (proteins) from interstitial tissue [39, 40], so the efficacy of this treatment modality in isolation is questioned, as it may not prevent fibrosis. In any case, the use of this therapy is recommended within the context of CDT, or at least in combination with MLD [41].

However, despite the favorable results found with the application of both kinesio taping in combination with an exercise program and MLD [32] and APCD in addition to usual care treatment [22] on the volume of external lymphedema, it is important to mention that both seem to be favorable for external lymphedema if applied as part of a multimodal treatment. Therefore, not all the effectiveness found [22, 32] could be attributed to APCD or the application of kinesio taping but to the sum of all the techniques used in each case.

Regarding internal lymphedema, neither the work of Atar et al. nor the work of Ridner et al. [22, 32] achieved a reduction. This could be due to the lack of effectiveness of the techniques described against this type of lymphedema, mainly due to the morphology and anatomical distribution of the different vessels and lymph nodes [42]. In this sense, Tritter et al. [43] in a pilot study with seven patients applied APCDs to improve post-radiotherapy laryngopharyngeal edema. After daily use (70% of cases) for approximately 6 months, no significant changes were obtained in the objective evaluation with endoscopic examination. However, patients reported substantial subjective improvement in dysphagia and dysphonia. These findings may warrant further formal investigation, and perhaps this is a hopeful conservative line of treatment for internal lymphedema in HNC patients.

Ozdemir et al. [21] demonstrated that both a supervised CDT program (MLD, plus compression therapy, plus exercise, and plus skin care) and a home program can improve some aspects of HNL. Other papers agree on the usefulness of self-administered treatment in obtaining a reduction in lymphedema [44, 45]. However, the presence of a specialized physical therapist performing the DLM and supervising the entire CDT protocol may lead to more extensive improvement [21, 46]. In this regard, the systematic review by Mullan et al. [17] of different types of HNL treatment (not just physical therapy) concludes that adherence to home-based interventions is generally poor. Thus, the treatment of choice would be the combination of face-to-face physical therapy and a supervised home program. Self-treatment would be recommended as the only approach in those cases in which conditions (lack of professionals, mobility difficulties, etc.) do not allow for in-person treatment.

Of interest is the early intervention performed by Tsai et al. [31]. This was the first study that worked with patients 7 to 10 days after surgery, achieving a significant ultrasound-assessed reduction in external lymphedema. Subsequently, Lemoine et al. [47] have applied a modified decongestive therapy in postoperative hospitalized patients, obtaining a reduction in lymphedema. These studies represent a first step toward the possibility of designing early hospital interventions that can reduce the effects of secondary HNL [48].

Overall, in our opinion, the various treatments administered in the studies examined must have had an influence on the fascial system of the face and neck. The lymphatic system is mostly contained at the level of the superficial fascia [42, 49, 50], and oncological therapeutic approaches (surgery and radiotherapy) cause damage to the connective tissue and lymphatic vessels. Physical therapy treatments that mobilize and release the fascial layers could benefit lymphatic drainage in lymphedema processes [51]. This issue has already been demonstrated in patients with breast cancer [52], although to our knowledge, it has not been raised in patients with HNC to date. In future research, it would be interesting to evaluate the changes at the fascial level of the face and neck.

Finally, the selection for quantifying the effect of adhering to the interventions was based on any of the studies included in this review using intention-to-treat analysis; two of them reported available data for all randomized participants [21, 31]. Per-protocol analysis attempts to quantify the undiluted effect of receiving treatment and can provide important information about the potential magnitude of treatment effects when patients adhere to the intervention [53]. Some of the studies failed to show adherence to the intervention [21, 22]. However, it should be taken into account that the treatment effect estimated from per-protocol analysis is frequently larger than the effect size estimated from the intention-to-treat [53]. We found the same overall RoB as did Jessica T. Cheng et al. [26].

As far as we know, sensitivity/precision analyses to recognize relevant databases have never been documented within this area (Online Resource 2). Medline recorded the highest sensitivity (75%). High sensitivity scores may reduce the chance of missing papers that are relevant. In contrast, Web of Science had a sensitivity of 25% and precision of 9.09, indicating that it was the most unsuccessful for use within this review. Logically, these finding are linked to the search string used in this review.

### Strengths and limitations

The strengths of this systematic review include the following: (1) the reporting was made according to the PRISMA guidelines; (2) the four studies included were published in the last 5 years, so they reflect the method of treatment and protocols most commonly used at present to treat secondary HNL; (3) a RoB assessment was included [54]; (4) sensitivity/precision analyses may inform future search strategies; (5) this review was registered prospectively in PROSPERO; and (6) this review aimed to include studies that presented high-quality evidence (RCT) to assess the efficacy only between interventions. Nevertheless, we know that the decision to include only this kind of design is justified to ensure clinical findings mainly regarding retrieved papers.

Despite the strengths mentioned above, some limitations should be taken into account: (1) choice of language (English/Spanish), thereby excluding possible relevant publications in other languages; (2) small sample sizes in all included studies; (3) predominance of patients with stage IB lymphedema (56.7%), thus making it difficult to extrapolate the results, as patients with higher stages might not improve with the treatments included in the present systematic review; (4) no homogeneity between the different studies in terms of therapies, treatment protocols, and duration of the interventions; (5) inconsistency in the methods of lymphedema assessment; (6) only one of the studies received a low overall risk of bias rating; and (7) high heterogeneity of studies, precluding a systematic quantitative analysis.

## Conclusion

Current evidence shows that APCD is an effective therapy for the management of external lymphedema and its associated symptoms, always as a complement to MLD and within the context of CDT, currently considered the gold standard in the management of lymphedema. Likewise, kinesio taping is positioned as a possible alternative to classic compression measures, and its application may be useful in cases of low tolerance to them. Finally, the lack of improvement in internal lymphedema with the therapies used, as well as the heterogeneity in the current literature regarding treatment times and protocols for the management of HNL, highlights the need for future lines of research aimed at clarifying this information to determine the most complete and precise approach to this prevalent sequela in patients with HNC.

## Supplementary Information

Below is the link to the electronic supplementary material.

Supplementary file1 (DOCX 13 KB)

Supplementary file2 (DOCX 17 KB)

## Data Availability

No datasets were generated or analysed during the current study.

## Abbreviations

AE: Adverse event
APCD: Automated pneumatic compression device
CDT: Complete decongestive therapy
CG: Control group
DLM: Draining lymphatic massage
EG: Experimental group
HNC: Head and neck cancer
HNL: Head and neck lymphedema
MDACC-HNL: MD Anderson Cancer Center Head and Neck Lymphedema
MLD: Manual lymphatic drainage
PRISMA: Preferred Reporting Items for Systematic Reviews and Meta-Analyses
RCT: Randomized controlled trial
RoB: Risk of bias
