# Supplementary material for: Lymphedema management in patients with head and neck cancer: a systematic review of randomized controlled trials on physical therapy interventions
Source: Support Care Cancer. 2025 Apr 26;33(5):420. doi: 10.1007/s00520-025-09438-1 (PMC12033101; doi:10.1007/s00520-025-09438-1)
Supplement: Supplementary file 1 — Supplementary file1 (DOCX 13 KB) [file 520_2025_9438_MOESM1_ESM.docx]

**Online Resource** 1**. Medline strategy search**

(Head and Neck Neoplasms[MeSH Terms] OR Head and Neck Neoplasms[All Fields] OR Head and Neck Neoplasm*[tiab] OR Cancer of Head and Neck[tiab] OR Cancer of the Head and Neck[tiab] OR Head and Neck Cancer[tiab] OR Upper Aerodigestive Tract Neoplasm*[tiab] OR UADT Neoplasm*[tiab] OR Head Neoplasm*[tiab] OR Neck Neoplasm*[tiab] OR Cancer of Head[tiab] OR Cancer of the Head[tiab] OR Head Cancer*[tiab] OR Cancer of Neck[tiab] OR Cancer of the Neck[tiab] OR Neck Cancer*[tiab]) AND (Physical Therapy Modalities[MeSH Terms] OR Physical Therapy Modalities[All Fields] OR Physical Therapy Modalit*[tiab] OR Physiotherap* (Techniques)[tiab] OR Physical Therapy Technique*[tiab] OR Group Physiotherap*[tiab] OR Physical Therap*[tiab] OR Neurological Physiotherapy[tiab] OR Neurophysiotherapy[tiab]) AND (Lymphedema[MeSH Terms] OR Lymphedema[All Fields] OR Lymphedema*[tiab] OR Milroy* Disease[tiab] OR Early Onset Lymphedema*[tiab] OR Hereditary Lymphedema*[tiab] OR Hereditary Lymphedema* 1[tiab] OR Hereditary Lymphedema* 1s[tiab] OR Hereditary Lymphedema Type I[tiab] OR Nonne Milroy Disease[tiab] OR Nonne Milroy Lymphedema[tiab] OR Nonne Milroy Meige Disease[tiab] OR Primary Congenital Lymphedema*[tiab] OR Congenital Familial Lymphedema[tiab] OR Congenital Hereditary Lymphedema*[tiab]) AND (randomized controlled clinical trial*[tiab] OR randomised controlled clinical trial*[tiab] OR randomized controlled trial*[Publication Type] OR randomised controlled trial*[Publication Type] OR randomized controlled trials as topic[MeSH Terms] OR randomized controlled trial*[All Fields] OR randomised controlled trial*[All Fields] OR clinical controlled trial*[tiab] OR controlled clinical trial*[tiab] OR clinical trial*[tiab] OR random allocation[tiab] OR randomly allocated[tiab] OR allocated randomly[tiab])
