# Supplementary material for: Lymphedema management in patients with head and neck cancer: a systematic review of randomized controlled trials on physical therapy interventions
Source: Support Care Cancer. 2025 Apr 26;33(5):420. doi: 10.1007/s00520-025-09438-1 (PMC12033101; doi:10.1007/s00520-025-09438-1)
Supplement: Supplementary file 2 — Supplementary file2 (DOCX 17 KB) [file 520_2025_9438_MOESM2_ESM.docx]

| Databases | Total hits  retrieved | Relevant  hits retrieved | NNR | Unique hits | Sensitivity | Precision |
| --- | --- | --- | --- | --- | --- | --- |
| Medline | 10 | 3 | 3.33 | 2 | 75 | 30 |
| Web of Science | 11 | 1 | 11 | - | 25 | 9.09 |
| Scopus | 3 | 1 | 3 | - | 25 | 33.33 |
| Cochrane | 8 | 2 | 4 | 1 | 50 | 25 |
| TOTAL | **32** | **4*** |  | | | |

Number asterisked (*) include total number of hits after duplicates removed.

NNR: Number Needed to Read (total hits retrieved/ relevant hits on a database).

Unique paper: relevant study retrieved from one database only.

Sensitivity: relevant hits retrieved / relevant hits retrieved TOTAL (%).

Precision: relevant hits retrieved / total retrieved from each database (%).

**Online Resource 2.** Sensitivity/precision analysis for each database.
